# Surgical management of head and neck squamous cell carcinoma metastatic to the parotid gland

**DOI:** 10.3389/fonc.2025.1579680

**Published:** 2025-05-27

**Authors:** Michał Gontarz, Marta Urbańska, Jakub Bargiel, Krzysztof Gąsiorowski, Tomasz Marecik, Grażyna Wyszyńska-Pawelec

**Affiliations:** ^1^ Department of Cranio-Maxillofacial Surgery, Jagiellonian University Medical College, University Hospital, Cracow, Poland; ^2^ Amethyst Radiotherapy Centre, Cracow, Poland

**Keywords:** parotid metastasis, secondary tumor, cutaneous squamous cell carcinoma, head and neck squamous cell carcinoma, carcinoma of unknown primary, elective parotidectomy

## Abstract

The parotid gland includes lymph nodes that may contain metastasis from the head and neck region as well as distant metastasis or metastasis from carcinoma/melanoma of an unknown primary (CUP/MUP). This study aimed to review the current literature to evaluate the role of surgical management of head and neck squamous cell carcinoma (HNSCC). A narrative review of the English language literature available in the PubMed and Embase databases from January 2010 to December 2024 was conducted to identify treatment methods and follow-up data for HNSCC metastatic to the parotid gland. This study provides a detailed overview of the histological and diagnostic imaging characteristics as well as the surgical and non-surgical procedures employed in the management of HNSCC metastases to the parotid gland. Furthermore, the management of CUP is outlined. The extent of the parotidectomy and concomitant neck dissection remains a topic of ongoing debate. The flow chart presented in this study may assist in decision-making regarding surgical treatment. Given that locoregional recurrence is the primary cause of mortality in HNSCC, surgical treatment is likely to be the most effective means of achieving a favorable outcome.

## 1 Introduction

The current classification of neck node levels designates level VIII as the parotid region, which includes four groups of lymph nodes: (I) subcutaneous pre-auricular nodes; (II) superficial intraparotid nodes; (III) deep intraparotid nodes; and (IV) subparotid nodes (1). Level VIII is responsible for draining lymph from a number of regions, including the forehead, temple, eyelids, conjunctiva, auricle, external acoustic meatus, tympanum, nasal cavity, root of the nose, nasopharynx, and eustachian tube (1). Efferent lymphatic drainage from level VIII flows to the deep cervical nodes in levels II and III, as well as to the superficial nodes associated with the external jugular vein (EJV).

As the global population ages and the incidence of skin cancer of the head and neck continues to rise, there is an increased risk of metastasis to the parotid gland. The highest rates of metastatic cutaneous head and neck squamous cell carcinoma (HNcSCC) of the parotid gland have been observed in Australia and New Zealand (2–4). The majority of metastatic malignancies observed in the parotid gland are regional metastases from HNcSCC and malignant melanoma. Furthermore, other regional metastases from primary tumors localized in the upper aerodigestive tract (UAT), conjunctiva, lacrimal gland, thyroid gland, and carcinoma of unknown primary (CUP) have also been observed (5).

Surgical intervention is the preferred treatment approach for managing metastatic disease in the parotid gland. The 2023 National Comprehensive Cancer Network (NCCN) guidelines for parotid gland metastases recommend superficial parotidectomy (SP) with ipsilateral neck dissection as indicated (6). However, controversies remain concerning the range of parotidectomy and neck management, especially in clinically N0 (cN0) neck cancer patients. Questions remain: Is elective parotidectomy in patients with high-risk HNcSCC and clinically negative lymph nodes? Is elective neck dissection (END) also indicated in this group of patients? Is SP sufficient for level VIII metastases? Should END be performed in cases with VIII-level involvement?

The aim of this study was to review the current literature to evaluate the role of surgical and non-surgical management in HNcSCC and non-HNcSCC metastatic to the parotid gland.

## 2 Methods

A narrative review of the English language literature available in the PubMed and Embase databases from January 2010 to December 2024 was conducted to identify surgical treatment methods and follow-up data for HNSCC metastatic to the parotid gland. The search was conducted using the following key terms: “head and neck squamous cell carcinoma,” “secondary parotid gland tumor,” “parotid metastasis,” “cutaneous squamous cell carcinoma,” “carcinoma of unknown primary,” “occult metastasis,” and “elective parotidectomy.” The reference section of each article was searched for additional potentially relevant publications. Only original and review articles were included in the analysis. Case reports were excluded from the study.

### 2.1 Diagnosis

#### 2.1.1 Imaging

**Figure 1:**
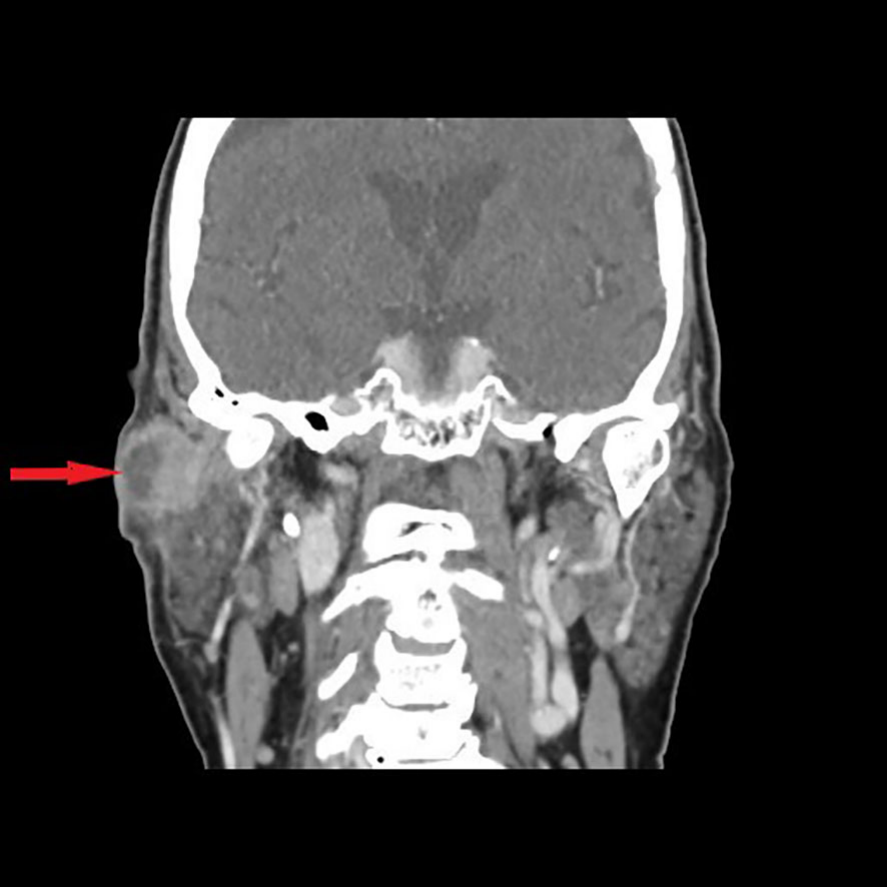
Contrast-enhanced frontal view CT scan shows a 29-mm mass with clear peripheral enhancement; the central part of the lesion is hypodense without a distinct enhancement—central necrosis (arrow), in the upper part of the superficial lobe of the right parotid gland (laterally to the retromandibular vein). Histopathological examination after parotidectomy and END (SND II, III, and EJV) revealed metastases in the two parotid lymph nodes without cervical lymph node involvement. The primary focus of the cSCC of the temple was excised 4 months earlier in another department.

Unlike other major salivary glands, the lymphatic system of the neck develops prior to parotid gland encapsulation. For this reason, the parotid gland contains lymph nodes, which should be considered metastatic drainage from regional (supraclavicular) or distant (infraclavicular) primary malignant tumors (7, 8). The cutoff point for the normal size of parotid lymph nodes in healthy adult patients, based on computed tomography (CT), is 5 mm, while nodes with a greater diameter should be considered as potentially pathologic (9). Clinicians should also bear in mind that in diagnostic imaging of the parotid gland, the round shape of the lymph nodes is typical, not pathological, similar to submental lymph nodes. The majority of metastases to the parotid gland are regional malignancies, especially those of the scalp and face. Proper differential diagnosis between metastatic and reactive lymph nodes plays a key role in treatment planning. However, diagnostic imaging is insufficient to confirm metastasis to the parotid gland. Currently, only a few studies have focused on diagnostic imaging of metastases to the parotid lymph nodes (8, 10). Kashiwagi et al. in CT and magnetic resonance imaging (MRI) observed well-defined margins in 61% and central necrosis in 18% of cases (**Figure 1**) (8). Kim et al. reported a higher percentage (57.6%) of central necrosis and cystic changes in metastases to the intraparotid lymph nodes based on CT and PET/CT findings. The study also compared imaging characteristics such as location in the parotid gland, maximum diameter, margins, central necrosis or cystic changes, and maximum standardized uptake value in 26 patients with intraparotid metastasis of non-cutaneous regional cancers and 20 patients with synchronous benign parotid tumors. Nevertheless, the study did not show any significant differences in imaging findings between the two groups of patients (10). Unfortunately, these results indicate that CT, MRI, and PET/CT features of metastasis to the parotid lymph nodes are non-specific and overlap with the imaging findings for benign and malignant parotid primaries or reactive lymph nodes (8, 10). Moreover, it should be noted that “false” positive, abnormal 18F-fluorodeoxyglucose (FDG) uptake in PET/CT is a typical feature of Warthin’s tumor and oncocytoma and may suggest the presence of metastatic lymph nodes or malignant primary tumor in the parotid gland (11–13).

### 2.2 Pathology

Fine needle aspiration cytology (FNAC) is widely accepted as the first step in the diagnosis of salivary gland tumors (14). The second edition of the Milan System for Reporting Salivary Gland Cytopathology (MSRSGC) represents a standardized, evidence-based reporting system for salivary gland FNAC. MSRSGC divides lesions into six categories, where each category has a suggested risk of malignancy ranging from <3% to >98% (**Table 1**) (15). Molecular studies may improve diagnostic assessment using FNAC. Examination of cytological samples with a comprehensive RNA-based panel can provide a more accurate assessment of the risk of malignancy (16). According to Horáková et al., metastasis to the parotid gland included only 1.6% FNAC salivary gland lesions (26 of 1,577 cases) with a sensitivity of 53%, specificity of 99.9%, positive predictive value of 87.5%, and negative predictive value of 98.9% (17). It is worth mentioning that a patient’s history of malignant disease and sufficient amount of cell material for immunocytochemical (ICC) staining play a crucial role for pathologists in the correct diagnostic procedure (17–19). Currently, the largest multicenter study of FNAC utility in the diagnosis of secondary malignancy of the salivary glands revealed SCCs in 47.3% of cases, followed by malignant melanomas in 36.4% (14). Moreover, in this study, metastases to the parotid gland were found in 171 (92.9%) patients, and the submandibular gland was involved in 13 (7.1%) patients (14).

**Table 1:** The second edition of the Milan System for Reporting Salivary Gland Cytopathology for FNAC of salivary gland tumors: implied risk of malignancy and recommended clinical management.

Diagnostic category	Risk of malignancy (%)	Management
I. Non-Diagnostic	15	Clinical and radiologic correlation/repeat FNAC
II. Non-Neoplastic	11	Clinical follow-up and radiologic correlation
III. Atypia of undetermined significance (AUS)	30	Repeat FNAC or surgery
IV. Neoplasm		
A. Neoplasm: Benign	<3	Surgery or clinical follow-up
B. Neoplasm: Salivary Gland Neoplasm of Uncertain Malignant Potential (SUMP)	35	Surgery
V. Suspicious for malignancy (SM)	83	Surgery
VI. Malignant	>98	Surgery

FNAC, fine needle aspiration cytologsy.

Core needle biopsy (CNB) is an alternative method for the histological diagnosis of salivary gland tumors (20, 21). However, to date, no published studies have specifically assessed the sensitivity and specificity of CNB for the identification of parotid gland metastases. A literature review by Hurry et al. on the diagnosis of salivary gland malignancies reported that the sensitivity of CNB ranged from 81.1% to 96.7% with an average of 91.4%. In comparison, the sensitivity of FNAC ranged from 59% to 93.5%, with an average of 74.1% (22). These findings demonstrate the superior sensitivity of CNB in detecting malignancy, which may be due to the larger volume of tissue obtained, allowing for a more comprehensive histological assessment including immunohistochemical staining. Furthermore, similar Milan-like categories can also be applied to core biopsy specimens. However, both FNA and CNB are unable to routinely demonstrate invasion, which limits their ability to definitively diagnose malignancy in some low-grade cancers (23).

**Figure 2:**
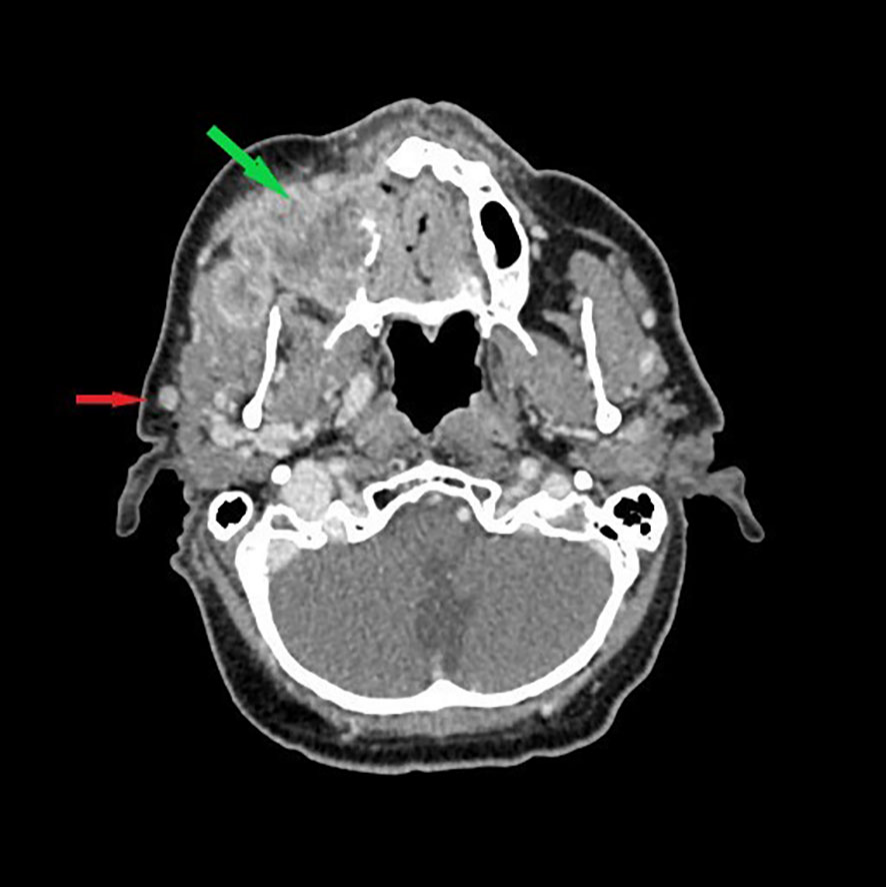
Contrast-enhanced axial view CT scan showing a 7-mm enlarged right parotid lymph node (arrow) in the patient with advanced SCC of the right maxilla (green arrow). During surgery, frozen sections of the parotid lymph node revealed no metastasis, only chronic inflammation, and parotidectomy was not performed.

Nevertheless, in the case of enlarged parotid lymph nodes with non-diagnostic FNAC/CNB and synchronous head and neck cancer, frozen section examination of the suspected parotid lymph node should be routinely performed during the surgical resection of the primary focus to determine the need for parotidectomy (**Figure 2**) (24).

### 2.3 Treatment

**Figure 3:**
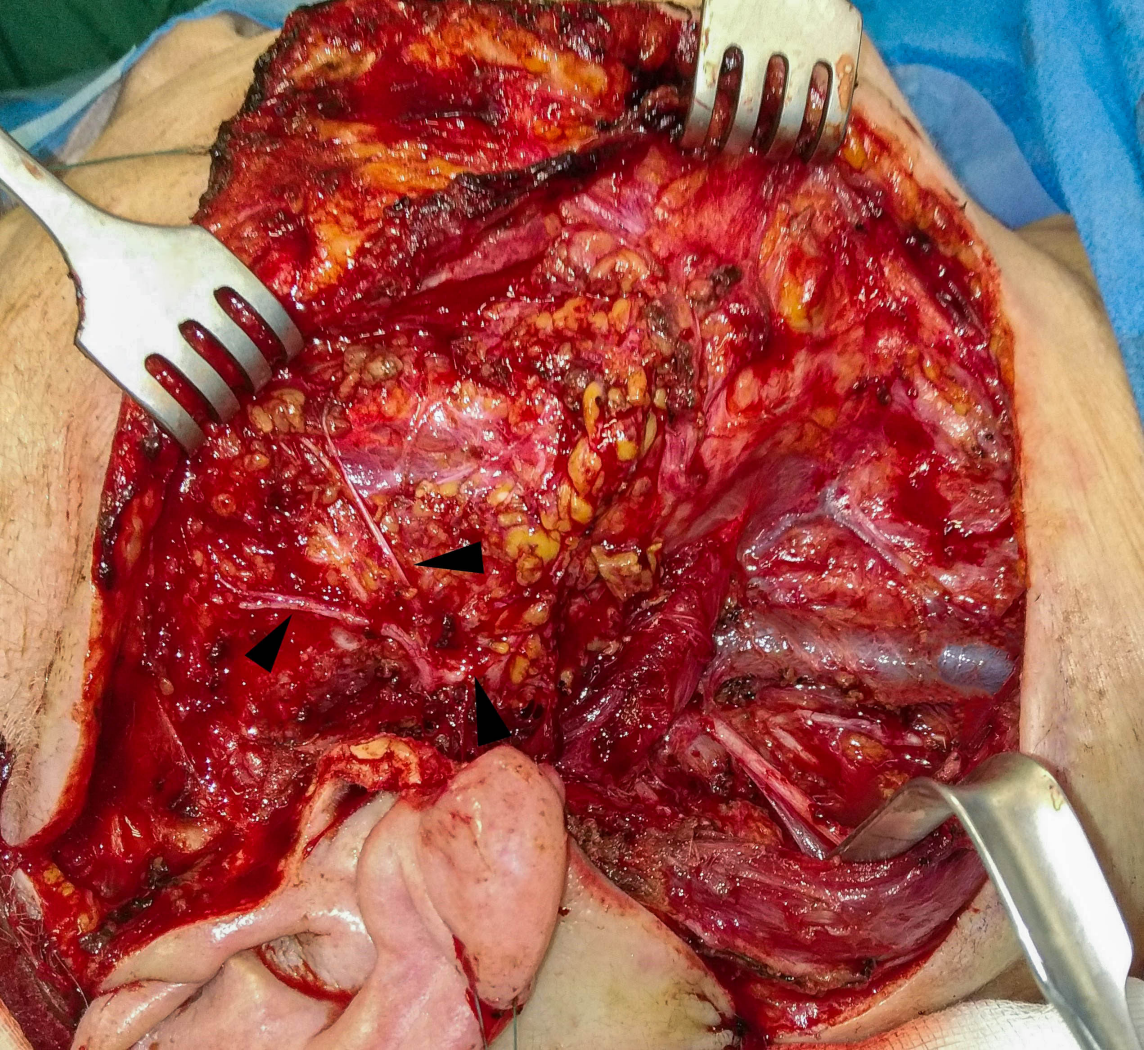
Clinical intraoperative view after parotidectomy with END (SND II, III, and EJV) with facial nerve preservation (arrows) due to cSCC metastasis in the parotid lymph nodes of the patients in **Figure 1**.

Surgical management is the treatment of choice for patients with salivary gland tumors. The facial nerve divides the parotid gland into superficial and deep lobes and is a hallmark of a surgeon during superficial parotidectomy (SP). The state of the art involves performing parotidectomy with preservation of the facial nerve. Most intraparenchymal lymph nodes were located in the superficial lobe. According to Ergün et al., a cadaver study revealed that 58 of 84 (69%) deep lobes of the parotid gland did not have lymph nodes (7). Considering this, it seems that SP is sufficient in the treatment of metastasis to the parotid gland (**Figure 3**). In contrast, Graham observed that all parotid lymph nodes are located lateral to the retromandibular vein, which lies deeper into the facial nerve (25). Therefore, SP following facial nerve dissection may cause residual disease in the parotid gland. The retromandibular vein is also a radiographic marker in diagnostic imaging that divides the parotid gland into superficial and deep lobes. Therefore, each tumor infiltrating deeper into the retromandibular vein, based on preoperative imaging, should qualify for total parotidectomy (TP) with facial nerve preservation (26).

#### 2.3.1 Head and neck cutaneous squamous cell carcinoma

The distribution of nodal metastasis of HNcSCC is mainly related to neck levels VIII and II (27–31). A multicenter study of 322 metastatic HNcSCC revealed parotid involvement in 81% (260/322), with parotid metastasis alone in 68% (217/322), and neck disease alone in 19% (62/322) (32). Multivariate analysis revealed that the independent risk factors for nodal metastasis in HNcSCC (high risk) are auricular site, tumor diameter >20 mm, tumor thickness >2 mm, moderate and poor differentiation, tumor recurrence, Clark level V and perineural/lymphovascular invasion (29, 33, 34). However, according to the NCCN, all SCC localized in the head and neck region are classified as high risk, as well as clinically poorly defined margins, recurrent tumor, immunosuppression, prior radiotherapy (RT) or site of chronic inflammation, rapid growth rate, neurological symptoms, depth of invasion (DOI) 2 mm–6 mm, and perineural involvement (6). In addition, NCCN stratification distinguishes very-high-risk SCC, which are cancers >4 cm in diameter, in clinical assessment. Histologically very-high risk SCC is poorly differentiated, or desmoplastic SCC with DOI >6 mm or invasion beyond subcutaneous fat, with tumor cells within the nerve sheath of a nerve lying deeper than the dermis or measuring ≥0.1 mm and lymphatic or vascular involvement (6).

##### 2.3.1.1 Elective parotidectomy

Wong and Morton recommended elective treatment in cases of HNcSCC, given that the risk of occult nodal metastasis exceeds 19% (35). However, occult metastasis to the parotid lymph nodes in patients with HNcSCC has been underestimated. Only a limited number of studies have evaluated occult metastases to the parotid gland and the necessity for elective parotidectomy in patients with HNcSCC (36–43). Elective SP was performed in most trials (6/8). The process of determining whether a patient is suitable for parotidectomy before surgery involves a combination of clinical and radiological evaluations. However, precise histological assessment of the HNcSCC subtype or other features, such as DOI, perineural involvement, or vascular invasion, is challenging when relying solely on incisional biopsy of the primary lesion. Preoperatively, only tumor diameters <4 cm and >4 cm could be directly used to classify patients into the very high-risk group for HNcSCC. A summary of the risk of occult metastasis in elective parotidectomy for HNcSCC with tumor diameters >4 cm and <4 cm is presented in **Table 2**.

**Table 2:** The risk of occult metastasis of HNcSCC in the parotid gland after elective parotidectomy with NCCN classification into high risk and very high-risk cancers based on the patient’s clinical examination (diameter of the primary focus).

First author	Type of elective parotidectomy	Number of occult metastasis following elective parotidectomy (%)		
		Diamater of HNcSCC <4cm	Diamater of HNcSCC >4cm	Total
Kadakia et al. (36)	SP	13/75 (17.3%)	10/18 (55.5%)	23/93 (24.7%)
Kadakia et al. (37)	SP	21/74 (28.4%)	18/30 (60%)	39/104 (37.5%)
Osborne et al. (38)	SP/TP	0/8 (0%)	0/3 (0%)	0/11 (0%)
Hoch et al. (39)	SP	0/9 (0%)	1/4 (25%)	1/13 (7.7%)
Chang et al. (40)	SP	6/43 (13.9%)	–	6/43 (13.9%)
Kampel et al. (41)	SP/TP	Not mentioned	Not mentioned	2/12
Horakova et al. (42)	SP	0/8 (0%)	0/4 (0%)	0/12 (0%)
Xiao et al. (43)	SP	Not mentioned	Not mentioned	11/55 (20%)
Total		**40/217 (18.4%)**	**29/59 (49.1%)**	**82/343 (23.9%)**

SP, superficial parotidectomy.

TP, total parotidectomy.

Based on the results presented, occult metastases were observed during elective parotidectomy in 49.1% of the cases, suggesting that elective parotidectomy is indicated for very-high-risk cN0 HNcSCC (>4 cm diameter). In addition, the best local control (LC) may be achieved with elective parotidectomy and END. END should include at least level II lymph nodes associated with the external jugular vein (EJV) at the anterior margin of the sternocleidomastoid muscle (43). Furthermore, in patients with positive findings in the neck lymph nodes (cN+), elective parotidectomy should be considered, given that the parotid lymph nodes serve as sentinel nodes in HNcSCC. Failure to address these issues could result in an elevated risk of residual tumor, which decreases the overall survival (OS) (41, 42). Sweeney et al. found occult neck lymph node disease in 16% of patients; however, 69% of these patients also had parotid lymph node metastasis. However, only 5% of these patients had occult neck node metastasis without parotid node involvement (44).

##### 2.3.1.2 Range of parotidectomy

In cases of HNcSCC with clinical and radiographical spread to the parotid gland, the problem is the range of parotidectomy. The NCCN guidelines for parotid gland metastases recommend parotidectomy (generally superficial) with ipsilateral neck dissection if indicated (6). However, is the SP sufficient to achieve the best local control?

Some studies suggest that LC and OS in SP and TP are comparable (5, 41, 45, 46). TP increases the time of surgery and may increase the risk of facial nerve paresis (42). In addition, facial asymmetry is more visible after TP and requires reconstruction with a periumbilical free abdominal fat graft (47).

However, Thom et al. reported a 26% rate of metastases to the deep parotid lobe in HNcSCC patients with superficial lobe involvement and 13% in patients with head and neck melanoma after TP (24). Due to the lack of a real barrier between the superficial and deep lobes of the parotid gland, metastases to the deep part of the gland may occur in approximately 20%–35.1% of cases (24). Skip metastasis to the deep lobe without superficial lobe involvement was not found (24). These results indicated very good LC after TP in 93% of the patients with HNcSCC. Wertz et al. compared the rate of parotid bed recurrence in patients after SP or TP due to metastatic malignant melanoma. Parotid bed recurrence was observed in 13% of the patients after SP and 0% after TP (p = 0.04). The frequency of complications after SP and TP was comparable (p = 0.99), the same as the facial nerve function (p = 0.32) (48). Studies by Olsen et al. support the need for TP in any preoperative or perioperative metastasis to the parotid gland (49, 50). Lymph node level VIII should be treated in the same manner as level II, which is divided by the accessory nerve into IIa and IIb, just as the parotid gland is divided by the facial nerve. Avoiding locoregional recurrence is what really matters because without life, there is no quality of life (50).

##### 2.3.1.3 Elective neck dissection

END in cN0 neck cancer patients with confirmed parotid metastasis of HNcSCC seems to be reliable in every case. According to Rotman et al.’s meta-analysis, 22.5% of occult metastasis was observed in specimens analyzed after END, performed in cases of VIII-level involvement (51). Moreover, the extent of END should be determined based on the primary lesion site. In primaries localized on the face, anterior part of the scalp, and external ear, END should contain levels II and III. The posterior scalp or neck require END in levels II, III, and Va (31). In contrast, clinically positive parotid and neck lymph nodes require TP with comprehensive neck dissection (CND, levels I–V).

Sentinel lymph node biopsy (SLNB) is an alternative less invasive procedure than elective parotidectomy/END. SLNB is widely used in head and neck melanoma staging and management; however, it is not well understood in HNcSCC, and its value is controversial. According to the literature, 0%–18.4% of SLNBs are positive for the presence of metastases (52–59). SLNB is a good method for nodal staging but has no prognostic value in assessing survival in high-risk HNcSCC (52, 57). Currently, it is unclear which patients with HNcSCC would benefit from SLNB. SLNB for HNcSCC is evolving, and prospective, randomized, multicenter trials are required to better understand and specify the indications for this procedure.

##### 2.3.1.4 Radiotherapy

Postoperative radiotherapy (PORT) should be considered in cases with one positive lymph node, less than 3 cm in diameter, after parotidectomy and neck dissection. In addition, PORT is recommended when histological examination has shown involvement of more than one lymph node, one metastasis with a diameter greater than 3 cm, or extracapsular extension (ECE). Definitive radiotherapy is an option for unresectable parotid lymph node metastases and may also be considered in cases of unacceptable esthetic outcomes or lack of patient consent for surgery (60, 61). Elective neck irradiation (ENI) is not routinely used without END or SLNB for the treatment of lymphatic drainage of the parotid gland, which might be considered in cases of very high-risk cancers such as T3 lesions in the periauricular or temporal localization, with DOI >6 mm, and perineural or lymphovascular invasion. Systemic treatment is also an option, e.g., immune checkpoint inhibitors, Cemiplimab or Pembrolizumab, when curative RT or surgery is not feasible (60–62).

##### 2.3.1.5 Summary guidelines

**Figure 4:**
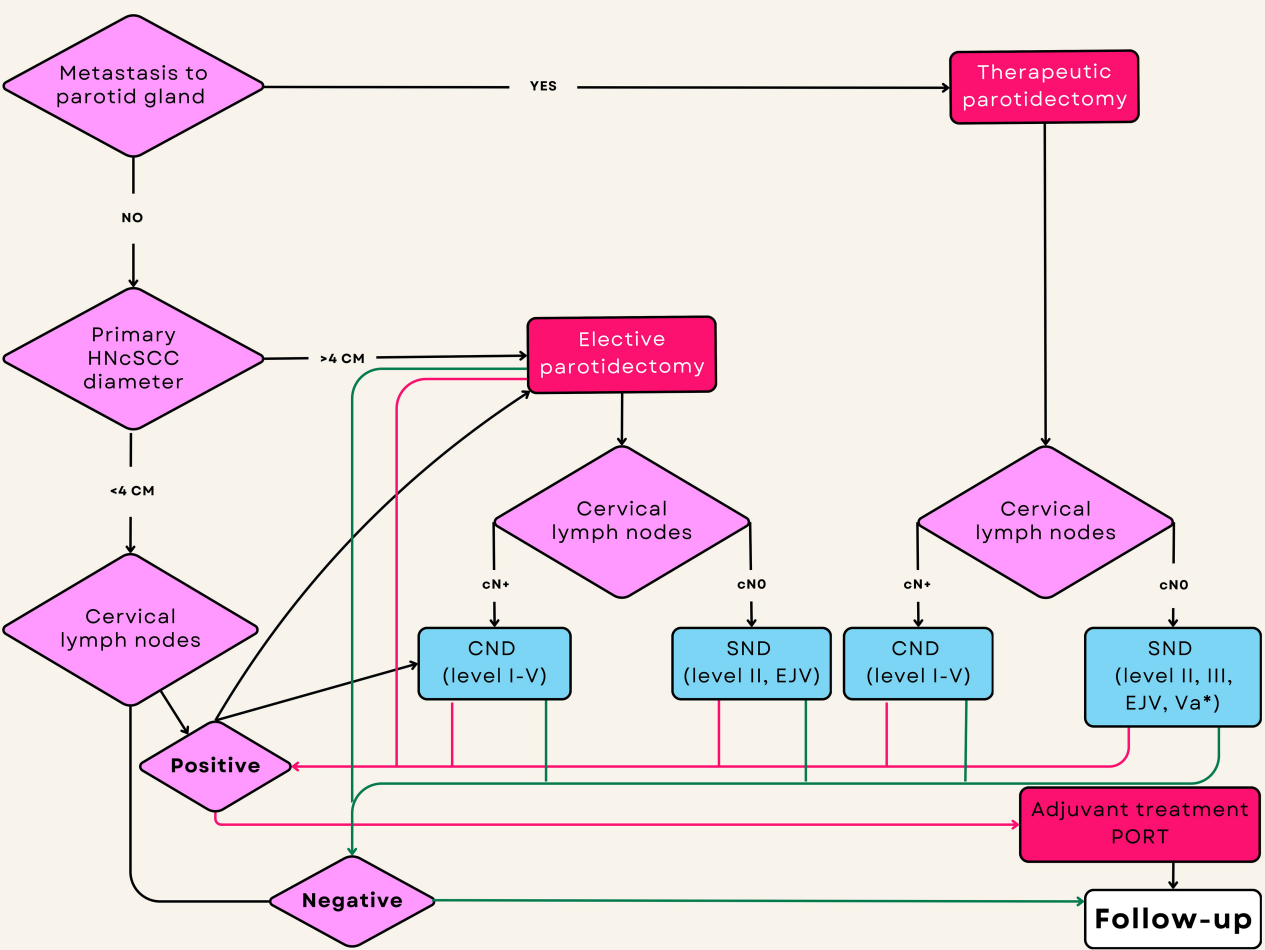
Flowchart showing decision making for surgical treatment of high-risk HNcSCC and indications for postoperative radiotherapy. The estimation of intraparotid lymph node metastasis is based on a combination of clinical, radiological, and FNAC/CNB findings. Green lines represent no metastases in the surgical specimen after neck dissection. Red lines represent histologically confirmed metastases after neck dissection. *Va is indicated for removal during selective neck dissection for primaries localized to the posterior scalp.

The proposed treatment planning workflow for high-risk and very high-risk HNcSCC at sites predisposed to metastasis to the parotid gland, based on the literature review, is shown in **Figure 4**.

Primary HNcSCC less than 4 cm in diameter without clinical and radiological evidence of metastasis to the parotid (P) and cervical (N) lymph nodes (P0N0) should be operated on for the primary lesion only, without elective parotidectomy and neck dissection. Close follow-up of the parotid and cervical lymph nodes should be maintained. On the other hand, very high-risk HNcSCC (>4 cm) P0N0 may require elective TP with END containing lymph nodes from level II and EJV due to the risk of occult metastases. Patients with P0N+ should be treated with CND and elective parotidectomy (TP) as the first metastatic basin. Patients with P+N0 require parotidectomy (TP) with END, including lymph nodes from II, III, EJV, and Va in the posterior scalp primaries because of the risk of occult metastases. Patients with P+N+ should be treated with parotidectomy (TP) or CND.

Elective neck irradiation (ENI) is a viable option for patients who are unsuitable for general anesthesia because of multiple comorbidities or who do not consent to surgical treatment of the cervical lymph nodes. While LC and OS outcomes are comparable between ENI and END, Xu et al. reported a poorer prognosis with ENI than with END when more than three metastases were present (63).

### 2.4 Upper aerodigestive tract—non-HNcSCC

Intraparotid lymph node metastasis from malignant tumors of the UAT is uncommon and associated with poor prognosis due to N stage advancement (64). SCC (non-HNcSCC) is the most common histological type of UAT cancer (10). In contrast to HNcSCC, parotid metastases of non-HNcSCC in nasopharyngeal and oropharyngeal/oral cancers are observed in only 0.4% and 0.8% of cases, respectively (64–66). Metastases from hypopharyngeal, laryngeal, and maxillary sinus cancers can be found (67). Nasopharyngeal and oropharyngeal cancers are thought to metastasize to the parotid gland via retropharyngeal lymph nodes (68). Moreover, Kim et al. and Olsen et al., observed parotid metastasis only in cases of dissemination into cervical level II (10, 66). According to their theory, parotid gland metastasis occurs due to the blocking of normal routes of lymphatic drainage and retrograde flow of cancer cells into the cervical level VIII from level II (10). In addition, prior surgery or radiotherapy might disturb anatomical lymphatic pathways, leading to the dissemination of cancer through alternative routes (7).

The treatment of choice for oral cancer is surgical resection of the primary tumor with neck dissection (selective or comprehensive). According to Olsen et al., during neck dissection, the tail of the parotid gland should be thoroughly inspected and resected as part of the surgical specimen (66). The risk of metastasis is less than 1%, and Warthin tumors might sometimes be misdiagnosed as metastasis in this region (10, 69). However, when metastasis is confirmed in the tail of the parotid gland, parotidectomy should be considered (67).

Nasopharyngeal, oropharyngeal, and hypopharyngeal cancers with metastases to the intraparotid lymph nodes require radical radiotherapy, usually combined with chemotherapy based on cisplatin, or when contraindications for that drug exist, anti-EGFR therapy should be considered. Surgery is reserved as salvage treatment. In pharyngeal cancer, nodal group VIII is not irradiated electively because of increased toxicity, leading to xerostomia and dysphagia (70).

In selected cases of very advanced pharyngeal cancer (cT4, cN2, and N3), neoadjuvant chemotherapy followed by radical radiotherapy/chemoradiotherapy could be a choice (70–74). This strategy does not improve OS but selects patients for radical treatment. In cases of complete remission (CR) or partial remission (PR) of nasopharyngeal and oropharyngeal cancers, radical radiotherapy or chemoradiotherapy is an option. For hypopharyngeal and laryngeal cancer with cartilage destruction (cT4), radical surgery followed by PORT is a preferable strategy (72). In cases with a lack of response or actual progression after neoadjuvant chemotherapy, palliative treatment is recommended, either radiotherapy or systemic therapy (71, 75).

In advanced nasopharyngeal cancer previously treated with combined chemoradiotherapy, adjuvant systemic treatment with cisplatin and fluorouracil is indicated. This is especially true for the appearance of EBV DNA in blood tests after combined radical treatment. Circulating EBV DNA is a negative prognostic factor for cancer recurrences (76).

### 2.5 Carcinoma of Unknown Primary

Initially, cases of CUP comprised 5%–10% of head and neck cancers, and approximately 75% of these were SCC. Usually, the primaries responsible for CUP of the head and neck are localized in the oropharynx. Nevertheless, thorough diagnostic work-up improves detection of primary and definitive CUP, accounting for approximately 1%–2% of head and neck cancers (77). True CUP of the parotid gland is rare. Approximately 10% of parotid metastases comprise initial cases of CUP or melanoma of unknown primary (MUP) origin (78). The pathologist plays an important role in the proper diagnosis of parotid CUP, which could guide further differential diagnosis to identify the primary lesion. In our opinion, SCC of the parotid gland cannot be considered CUP. Initially, parotid SCC should always be classified as a secondary tumor. For this reason, thorough anamnesis of previous treatments of skin and UAT cancers is very important. In the case of parotid SCC, pathologists should perform p16/HPV immunohistochemical staining and/or EBV DNA. Positive p16/HPV testing suggests naso-oropharyngeal SCC primary, but negative should be considered a non-naso-oropharyngeal site of the primary skin (77). Positive results for EBV and negative results for HPV staining might also suggest nasopharyngeal cancer. This information is very important for including appropriate region of the pharynx in radiotherapy. In the case of SCC, CUP curative radiotherapy should be considered as the primary treatment with elective nodal irradiation (71). Surgery is the method of choice for suspected skin cancer. Moreover, in cases of multifocal skin cancers, the exact finding of the primary site of SCC metastasis is almost impossible. However, only negative clinical examination, diagnostic imaging, and history of skin, UAT, or other sites of cancer lead to the classification of parotid SCC as a primary lesion. The same problem is associated with small cell carcinoma, adenocarcinoma, or clear cell carcinoma, where the primaries should be examined in the lung, breast, gastrointestinal tract, or kidney. Nonetheless, the present study by Bishop et al. did not identify a unifying genetic alteration characteristic of the primary SCC of the parotid gland. Instead, their meticulously curated series revealed cases with genetic profiles indicative of keratocystoma, porocarcinoma, cutaneous SCC, or SCC originating at other sites. Consequently, this study was unable to definitively confirm or rule out the existence of SCC arising from the parotid gland (79).

## 3 Study limitation

Because all articles were reviewed and selected manually, the number of articles analyzed was limited. The majority of the articles identified were retrospective studies with a single-institution experience. A meta-analysis would provide a more objective assessment of treatment methods. However, due to the wide range of problems related to the topic of elective/therapeutic parotidectomy, range of parotidectomy and neck dissection, and SLNB, a meta-analysis was not performed in favor of the narrative nature of this review. In addition, only eight trials that addressed elective parotidectomy for HNcSCC. Only one study was prospective, and the rest were retrospective. Another problem is that most patients (197/343; 57.4%) who underwent elective parotidectomy were treated at a single center in Texas, USA. The lack of clinical trials on head and neck SCC metastasizing to the parotid gland also does not allow more precise surgical guidelines to be defined. The most important problem that remains unresolved is the lack of universal guidelines for the management of secondary parotid tumors. These are relevant clinical issues that should be further investigated in future prospective, randomized, and multicenter trials.

## 4 Conclusions

In conclusion, this narrative review summarizes and synthesizes the problem of surgical treatment of metastasis to the parotid gland of HNSCC, which represents a diverse group of neoplasms. Moreover, the range of parotidectomy and concomitant neck dissection remains debated. The presented flow charts may facilitate decision-making for surgical treatment. Because locoregional recurrence is the main cause of death in HNSCC patients, the proposed surgical treatment is the best method to achieve favorable locoregional control. In addition, surgeons should bear in mind that the presented flow charts are based mostly on non-randomized clinical case controls and are not generally accepted international guidelines. Currently, the range of parotidectomies and neck dissections is limited. SLNB seems to be beneficial in patients with skin cancers metastasizing to the parotid gland, but there is still a lack of specific indications, especially for HNcSCC. Moreover, most patients with secondary tumors in the parotid gland are elderly and have multiple comorbidities. For this reason, therapy decisions should be based on geriatric examination and individual assessment of the patients, and minimally invasive therapy should be considered.
